# Telemedicine for Patients with COVID-19: A Telehealth Experience in the Elderly at a Center in Southern Brazil

**DOI:** 10.1089/tmr.2024.0009

**Published:** 2024-07-18

**Authors:** Natalia Contini, Samantha Lia Ziotti Bohn Soares, Asdrubal Falavigna

**Affiliations:** ^1^ Physicians graduated from the University of Caxias do Sul (UCS), Caxias do Sul, Brazil.; ^2^ Neurosurgery department at the University of Caxias do Sul, Rio Grande do Sul, Brazil.

**Keywords:** telemedicine, COVID-19, telehealth, e-health

## Abstract

**Background:**

Telemedicine has shown benefits in continuous care during the COVID-19 pandemic. This article discusses its practice in elderly patients with COVID-19, considering its limitations and benefits.

**Methods:**

Patients with COVID-19, aged 60 years or older, were followed up through phone calls three times a week for 10 days at the Telemedicine Section of the Clinical Center of the University of Caxias do Sul (UCS) in the south of Brazil. The outcomes evaluated were referrals to hospital, basic health unit (BHU)/emergency care unit (ECU), and psychology and physiotherapy services; instructions about vaccination, isolation period, tests for COVID-19, taking a specific medication, and measuring oxygen saturation; guidance to family members; and avoiding going to hospitals.

**Results:**

A total of 64 patients were followed up, the mean age was 69.28 years and 15.62% had at least one comorbidity. Among the patients, 7.81% were instructed about the vaccine, 23.43% about post-diagnostic tests, 25% about medication, 62.5% about isolation, 31.35% received guidance on saturation monitoring and 28.12% received guidance for family members, and 3.12% were referred to the hospital and 7.81% to the BHU/ECU (*n* = 5/64). Physiotherapy and psychology services were indicated for 4.68% of patients each, hospital visits were avoided in 31.25% and 93.75% recommended telemonitoring.

**Discussion:**

In this experience, it is suggested that the telehealth service maximizes patient care and the health care effectiveness for patients with COVID-19. Furthermore, the sample studied showed good adherence and suggested the need for more guidance than face-to-face consultation.

## Introduction

Overwhelming evidence from around the world suggests that age itself is the most significant risk factor for severe COVID-19 disease and its adverse health outcomes.^1^ Data reported by the Centers for Disease Control and Prevention (CDC) demonstrate significantly higher rates of hospitalizations, intensive care unit (ICU) admissions, and deaths secondary to COVID-19 among older adults (> 65 years of age) than younger age groups, with the highest percentage of severe outcomes among persons ≥85 years of age.^2^

Many underlying comorbidities also have been identified in the progression of COVID-19 into a severe and critical stage, including hypertension, heart disease, diabetes, obesity, chronic obstructive pulmonary disease (COPD), heart, liver and kidney diseases, tumors, and immunodeficiencies.^3,4^ The high alveolar viral load at the onset of those conditions is closely correlated with disease progression, especially leading to hypoxemia.^5–9^

Thus, it is important to pay close attention to these patients, and telephone triage to determine need for further evaluation is the preferred initial management approach.^10^ Avoiding unnecessary face-to-face medical visits, exposing other patients and health professionals to the virus, clarification of doubts, guidelines, referrals to health services, continuous evaluation, and prevention of complications are benefits of the practice of telemedicine in the pandemic. Furthermore, it proposes a better cost/benefit, enabling access to remote areas and communities where there is a shortage of doctors. Given the high risk of transmission of the virus through person-to-person contact, telemedicine can be beneficial in reducing direct contact and monitoring patients.^11^

Several studies have reported the burden of mental health problems among the general population during COVID-19.^12,13^ Therefore, psychiatric and psychological services also play a pivotal role in the overall disease control.^14^ In times of global lockdown, mental health services should focus on providing help through telemedicine approaches.^15^ A study from India showed that more than 80% of participants felt the need for professional help from mental health experts to deal with emotional issues and other psychological issues during this pandemic.^12^

Rehabilitation is also important for patients who have recovered from the acute respiratory effects of COVID-19 and can be offered remotely.^16^ After discharge from hospital, some systemic dysfunction (such as respiratory function, muscle weakness, neuropathy, and psychological disorder) may still occur.^17^ Data from studies demonstrated impairment in lung function in up to 23% of the patients at 1-year follow-up, as well as a reduction in exercise capacity.^18,19^

Recognizing the reduction in indirect costs of care that telemedicine can provide, along with a high patient satisfaction, encourages the adoption of these services in future models of health service delivery. The current pandemic has rapidly accelerated the transition to telemedicine and provided medical schools with the opportunity to prepare the students to participate and develop skills to assist patients remotely.

This article presents an experience of monitoring patients aged 60 years or older with COVID-19, seen at the Telemedicine Section in the Clinical Center of UCS (CECLIN-UCS) in the south of Brazil. The study participants were followed up through phone calls. The teleconsultation was intended to clarify doubts, inform, and monitor mild cases. In the videoconference, cases with an unfavorable progression, warning signs, and anxious patients were monitored.

The objective was to describe the teleconsultation experience amid elderly patients with COVID-19 to assess their adaptation to non-face-to-face consultations. We sought to investigate whether telephone follow-ups can be allies in improving health care. We also emphasize that telemedicine has become a great complement to face-to-face consultations, without replacing them.

## Materials and Methods

### Study design

Patients aged 60 years or older with COVID-19, who were not hospitalized, were monitored between January and February 2021 at the Telemedicine Section in the Clinical Center of UCS (CECLIN-UCS). They were divided into two groups, each one followed by a medical student for 10 days, all through phone calls three times a week without control group. Two medical students, always under the supervision of a physician, performed the monitoring. This study was performed in line with the principles of the Declaration of Helsinki and electronic informed patient/participant consent was obtained. The study was approved by the Medical Ethics Committee of the University of Caxias do Sul.

Before calling the patient, the clinical data were reviewed to pay attention to risk factors for an unfavorable evolution of COVID-19. In addition, the date of onset of symptoms and, for asymptomatic patients, the date of the positive test for Severe Acute Respiratory Syndrome Coronavirus 2 (Sars-CoV-2) were collected to provide guidance on the isolation period. The connection was established through a phone call and the consultation began with greetings, introducing herself or himself, clarifying the reason for the contact, and guaranteeing security and privacy.

Afterward, the phone number and the audio were checked by asking the patients if they could hear. The medical assessment started with open-ended questions, followed by an exploration of the main symptoms of COVID-19 and warning signs, considering the patient’s comorbidities. The patient was asked about the characteristics of symptoms and changes in the previous pattern, the medications in use, and the results of imaging examinations. If the patient had a thermometer and/or oximeter at home, their body temperature and/or oxygen saturation were reported.

Patients with mild to moderate disease based on signs and symptoms were instructed to discontinue the isolation when at least 10 days had passed since symptoms first appeared, at least one day (24 h) had passed since the resolution of fever without the use of fever-reducing medications, and symptoms had improved (e.g., cough, shortness of breath). For asymptomatic patients, the isolation period of 10 days was counted from the date of their first positive COVID-19 test if there was no evidence of subsequent illness. If the symptoms developed, a symptom-based strategy was used, according to established recommendations.^20^

This information was recorded on individual spreadsheets, indicating the symptoms of COVID-19 presented and additional information. Then, orientations about vaccination, isolation period, tests for COVID-19, taking a specific medication, measuring oxygen saturation, and guidance to family members were carried out. Referrals to the basic health unit (BHU)/emergency care unit (ECU), and psychology and physiotherapy services were also performed if indicated. A video consultation was conducted in the presence of warning signs (dyspnea, chest pain/pressure, dizziness, falls, hypotension, mental status change, cyanosis, and hypoxemia) or for clarification. In addition, the patients were referred to a face-to-face service according to the symptoms presented.

### Patient eligibility

The initial information on the patients was provided by the municipal health department, including notification date, name, mother’s name, date of birth, age, sex, municipality of residence, the neighborhood of residence, criterion, workplace, epidemiological link, fever, respiratory symptoms, other symptoms, comorbidities, hospitalization, date of first symptoms, the positive test, collection date, symptoms/collection time, outcome, type of test used in diagnosis, and contact phone. The inclusion criteria were 60 years of age or older, living in Caxias do Sul, RT-PCR or antigen test positive for SARS-CoV-2, and in-home isolation. The exclusion criteria were death before starting telemonitoring, wrong phone number, and never answering the phone calls.

### Variables analyzed

Based on this prospective study design, the outcomes evaluated were referrals to hospital, to BHU/ ECU, and to psychology and physiotherapy services. In addition, instructions were given about vaccination, isolation period, tests for COVID-19, taking a specific medication and measuring oxygen saturation. The number of people who avoided going to the hospital and instructions given to family members were also counted.

### Statistical analysis

Data were collected using the Microsoft Excel 2019 software program. The categorical variables are presented in percentage. The data presented are not publicly available or accessible.

## Results

**FIG. 1.**
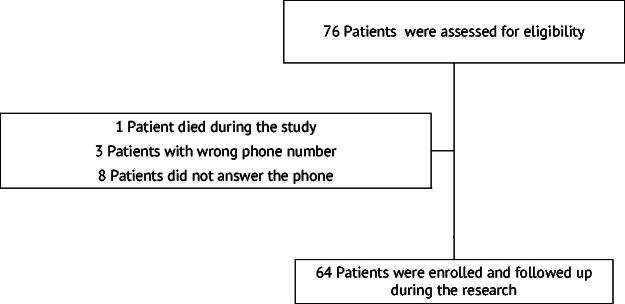
Reasons for excluding patients.

Initial data from 76 patients were provided by the municipal health department. However, one patient died before starting monitoring, eight never answered the phone calls, and three had wrong phone numbers. As a result, 64 patients met the inclusion criteria and were followed by the telemedicine service (Fig. 1). In this study, the patients were 60 years of age or older with a mean age of 69.28 years. Other risk factors for severe COVID-19 were also highlighted. Four patients had only heart disease, two only diabetes mellitus, one pulmonary emphysema, one immunosuppression, one diabetes and hypertension, and one heart disease and diabetes. As a result, 15.62% (*n* = 10/64) had at least one comorbidity (Table 1).

**Table 1.** Demographic Characteristics of the Participants

Characteristics	Percentage
Sex	
Male	57.81%
Female	42.19%
Age Group	
60–79 years	98.44%
Older than 80 years	1.56%
Comorbidities	
No comorbidity	84.38%
One or more comorbidities	15.62%

The follow-up suggests that COVID-19 patients mainly need guidance. In this study sample, 7.81% were instructed about the vaccine (*n* = 5/64), 23.43% about post-diagnostic tests (*n* = 15/64), 25% about medication (*n* = 16/64), and 62.5% about isolation (*n* = 40/64). In addition, 31.35% received guidance on saturation monitoring (*n* = 20/64) and 28.12% received guidance for family members (*n* = 18/64). In this sample, 6.35% of patients (*n* = 4/64) were referred to video calls, due to an unfavorable progression, warning signs, or anxiousness.

There were also referrals to face-to-face services based on exacerbation of COVID-19 symptoms, although to a lesser extent than telephone guidance. Around 3.12% were referred to the hospital (*n* = 2/64) and 7.81% to the BHU/ECU (*n* = 5/64). Moreover, multidisciplinary physiotherapy and psychology services were indicated for 4.68% of patients each (*n* = 3/64). In that telemonitoring, hospital visits were avoided in 31.25% of the cases (*n* = 20/64) (Table 2).

**Table 2.** Interventions during Follow-up of the Participants

Interventions	Percentage
Guidance	
About isolation	62.50%
About Saturation monitoring	31.35%
For family members	28.12%
About medication	25.00%
About post-diagnostic tests	23.43%
About the vaccine	7.81%
Referrals	
Basic health unit/emergency care unit	7.81%
Hospital	3.12%
Multidisciplinary services	
Physiotherapy	4.68%
Psychology	4.68%
Face to face consultations avoided	31.25%

After follow-up by telephone, the patients answered a satisfaction questionnaire, 93.75% of them recommended the monitoring (*n* = 60/64). The reasons for not fully recommending the consultation would be telephone contact made with different people, contact made with the patient’s family, connection problems, and technological limitations.

## Discussion

Although all patients in our sample were aged 60 years or older, the mean age was only 69.28 years. Early data from China demonstrated that the case fatality ratio of COVID-19 increases with age, from 0.4% or lower in patients in their 40s or younger, 1.3% among those in their 50s, 3.6% in their 60s, 8% in their 70s, to 14.8% in their 80s or older.^21,22^ In our study, only one death was recorded in a 96-year-old patient, as few patients were older than 80 years.

Only 15.62% had at least one comorbidity, with heart disease being the most common (7.81%), followed by diabetes (6.25%). Immunosuppression, hypertension, and emphysema were presented by only one patient each. Diabetes is a common comorbidity in COVID-19 patients^23^ and was suggested as a risk factor for severe disease or death, and is associated with a higher rate of ICU admissions.^24,25^ Similarly, the presence of heart disease is an important risk factor regarding the prognosis of COVID-19 patients progressing with acute myocardial injury.^26^

COPD and HIV are not associated with increased susceptibility to SARS-CoV-2 infection. However, once the patients develop the disease, they also have an elevated risk of hospitalization, ICU admission, severe disease, and mortality.^3,27^ Arterial hypertension was also more frequently observed in severe COVID-19 patients compared to not severe patients.^28,29^

It is important to note that the mean age was 69.28 years, only 15.62% had at least one comorbidity, and all participants were initially in home isolation. Thus, it is possible that patients at a higher risk of an unfavorable progression of COVID-19 may have been excluded from our study. A plausible reason is that they could already be hospitalized, given the greater severity of the condition. As a result, few referrals were made for face-to-face care, 3.12% being referred to the hospital and 7.81% to the BHU/ECU.

Many people are experiencing an ongoing, pervasive sense of loss: the tragic deaths and threatened loss of loved ones; the loss of physical contact with family members and social networks; and the loss of jobs, financial security, and livelihoods.^30^ Some patients from the sample were going through a period of mourning, due to the loss of a family member from COVID-19. Thus, psychology services were indicated for 4.68% of patients. This is a small number, compared to previous data. One hypothesis is that, because psychological care was offered by telephone, few expressed an interest in this modality, for privacy reasons.

This monitoring allowed early identification of persistent symptoms and disability, as well as early referral for physiotherapy in 4.68% of the patients. There are a variety of sequelae associated with the viral illness and with a prolonged stay in the ICU, possibly including mechanical ventilation.^16^ As few patients in our sample required ICU admission, there was a lower proportion of referrals to physiotherapy.

Due to this profile of patients in our study, telemedicine proved to be an excellent tool to avoid unnecessary face-to-face medical consultations as hospital visits were avoided in 31.25% of cases, preventing oversaturation of health facilities. This study showed that most patients with COVID-19 need guidance, especially about the isolation period (62.5%). Another common doubt was about the need for post-diagnosis examinations (23.43%). They were instructed to not repeat the test unless their job required it.

For most immunocompetent patients with COVID-19, a non-test-based strategy should be used to determine when precautions can be discontinued. This approach is supported by both the CDC and the World Health Organization. Some patients have persistently positive PCR testing for SARS-CoV-2 for weeks to months after resolution of symptoms, and this can unnecessarily prolong the need for infection prevention precautions and isolation, since prolonged viral RNA shedding after symptom resolution is not clearly associated with prolonged infectiousness.^31^

Instructions were also given to family members for 28.12% and symptomatic medications were indicated for 25% of the patients, according to symptoms presented and indications on a case-by-case basis. Measurement of oxygen saturation was recommended for 31.35% of patients who did not have a pulse oximeter at home and had dyspnea. Those who had a pulse oximeter at home reported the values obtained and a face-to-face assessment was indicated when oxygen saturation reached values less than or equal to 94%.

The ease of use of this device, combined with the large burden of illness in COVID-19 and the risks of silent hypoxia, make it a reasonable solution to monitor at-risk individuals.^32^ However, the cost restricts its wide use in Brazil. Furthermore, only 7.81% of the patients were advised about the vaccine, as vaccination was starting in the region and the study patients still did not include the vaccination group, as at the time only health professionals were being vaccinated.

Although all the patients in our sample were aged 60 years or older, contrary to the common belief that the elderly are reluctant to use technology, a high level of satisfaction was observed, as 93.75% would recommend the monitoring to someone else.^33^ However, most patients needed help from a younger family member to deal with technological issues, particularly when conducting a video call. This monitoring was performed by two medical students, always under the supervision of a physician. Perhaps the adoption of telemedicine in medical education will help future doctors prepare for the current era of COVID-19 and the upcoming pandemics.^34,35^

## Conclusions

Telemedicine was implemented to adhere to social distancing guides for infection control, allow continuous care, and reduce costs. In our experience, the telehealth service suggested maximizing patient care and health care effectiveness for COVID-19 patients. The study population consisted of elderly people who, although not so familiar with technology, adhered to telemonitoring and demonstrated a high level of satisfaction. Regarding conduct, patients with COVID-19 in isolation in this study required more guidance than referrals to hospitals. Therefore, guidance can be provided through telemedicine, avoiding the overload of health services due to misinformation. In addition, the COVID-19 pandemic has provided medical schools with the opportunity to incorporate telemedicine training into the curricula in a timely and practical manner. It is important to highlight that the follow-up presented was carried out through telephone calls, losing sight of the patient’s appearance and clinical signs, which could be assessed through video calls. Furthermore, the sample was small and only patients with COVID-19, aged 60 years or older, were included. As such, the conclusions cannot be generalized.

## Abbreviations Used

BHU: Basic Health Unit
CDC: Centers for Disease Control
CECLIN-UCS: Clinical Center of the University of Caxias do Sul
COPD: Chronic Obstructive Pulmonary Disease
COVID-19: Corona Virus Disease 2019
ECU: Emergency Care Unit
HIV: Human Immunodeficiency Virus
ICU: Intensive Care Unit
PCR: Polymerase Chain Reaction
RNA: Ribonucleic Acid
RT-P CR: Reverse transcription polymerase chain reaction
SARS-CoV-2: Severe Acute Respiratory Syndrome Coronavirus 2
UCS: University of Caxias do Sul
